# Valorization of *Caragana korshinskii* Kom. using cooperative *Aspergillus oryzae* and *Saccharomyces cerevisiae* to produce fermented feed protein

**DOI:** 10.1186/s40643-025-00968-4

**Published:** 2025-11-05

**Authors:** Sasa Zuo, Jing Su, Fuqiang Zhang, Shuying Yu, Xiaohui Cao, Chuncheng Xu

**Affiliations:** Department of Agricultural Engineering, College of Engineering, China Agricultural University, (East Campus), 17 Qing‑Hua‑Dong‑Lu, Haidian District, Beijing, 100083 People’s Republic of China

**Keywords:** *Caragana Korshinskii* kom., Feed protein, Solid-state fermentation, Optimization, Proteomics

## Abstract

*Caragana korshinskii* Kom. represents a substantial biomass resource that can be converted into feed protein via microbial fermentation. This study aimed to improve the nutritional value of *C. korshinskii* through strain screening and substrate optimization. Amino acid content and in vitro digestibility were systematically investigated. Astral-DIA proteomics was employed to compare protein enrichment mechanisms underlying screened microbial involvement in substance conversion. The *Aspergillus oryzae* and *Saccharomyces cerevisiae* co-culture increased the true protein content of the optimized substrate by 50.6% to 67.9%, while the highest nitrogen conversion ratio (69.5%) was achieved with low-level supplementation of (NH_4_)_2_SO_4_. The relative abundances of hydroxyproline and lysine content increased by more than twice in the mixed fermentation. Proteomics analysis identified 291 differentially expressed proteins in the mixed culture versus *A. oryzae* alone, enriched in ribosome biogenesis; valine, leucine and isoleucine biosynthesis; galactose metabolism; amino acids biosynthesis and sulfur relay system. This study provided guidance for the high-value utilization of *C. korshinskii* and elucidated the differential protein enrichment pathways between *A. oryzae*, *S. cerevisiae* and their cocktail in utilizing *C. korshinskii*.

**Graphical abstract:**

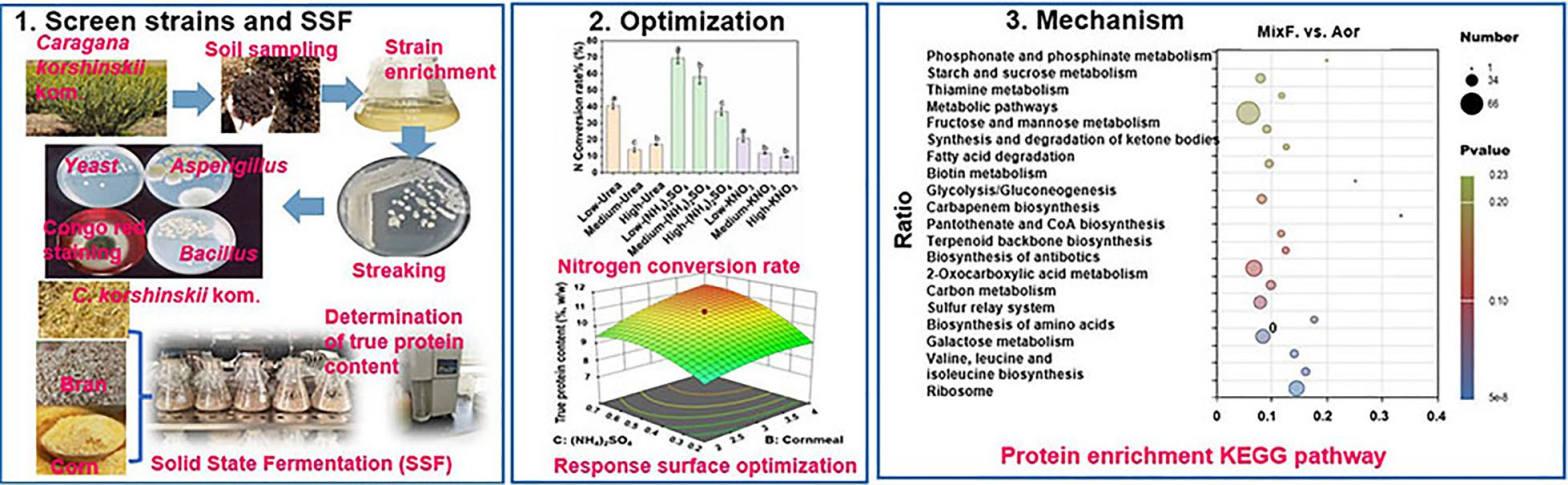

**Supplementary Information:**

The online version contains supplementary material available at 10.1186/s40643-025-00968-4.

## Introduction

The increasing global population and demand for meat and dairy products have driven the expansion of animal husbandry. This demand results in a lack of feed resources, particularly protein feed. The per capita demand for animal protein in China is estimated to increase by 22% to approximately 46 g/d by 2035 (Liu et al. 2023). Therefore, it is important to alleviate the food crisis and ensure food security by developing non-conventional feed resources, finding ways to reduce and substitute soybeans, and relieving the lack of protein feed resources. *Caragana korshinskii* Kom. is a common cultivated species of the *Caragana* genus. The extensive planting of *C. korshinskii* for grazing prohibition and sand control has led to the annual generation of over 4 million tons of waste (Bai et al. 2023; Ke et al. 2024). The leaves and blossom of *C. korshinskii* have high nutritional value and economic benefits as crude feed for ruminants after processing. However, the large biomass of pruned branches of *C. korshinskii* contains more than 70% lignocellulose, and lower content of protein (Du et al. 2024). Consequently, *C. korshinskii* should be processed and modulated to improve its feed quality.

To date, a few studies have been conducted to improve the nutritional value of *C. korshinskii*, including ensiling, enzyme hydrolysis and white-rot fungal treatment (Du et al. 2024; You et al. 2022; Zhang et al. 2023). However, there are still challenges in these methods, for example, low concentration of water-soluble carbohydrates, high cost, and easily be contaminated (Ke et al. 2024; Zuo et al. 2018b). Filamentous fungi, such as *Aspergillus* sp. are widely used in fermented foods and feeds. Yeasts have been used to produce single-cell proteins with an abundant mycoprotein content. Moreover, some bacteria, such as *Bacillus*, also can degrade lignocellulose (Wang et al. 2024). The use of these strains in solid-state fermentation (SSF) is inexpensive, easily operated, and reacts mildly; therefore, it is suitable for selecting these strains for feed protein production. In addition, fermentation conditions and substrate compositions also can significantly affect the protein enrichment (Raimbault 1998).

Synthetic ecosystems with intermediate complexity and high controllability are more resilient and experience less metabolic burden than monocultures, making them suitable for various biotechnological applications (Liu et al. 2022). Proteomic analysis is currently the most powerful method for identifying proteins in microorganisms and understanding the mechanism of protein expression involved in complex biological pathways (Sethupathy et al. 2021). A few studies have compared the different secretome proteins between solid-sate and submerged fermentation with different *Aspergillus* species (Ma et al. 2021; Salgado-Bautista et al. 2020). The application of proteomics to compare the growth of *Saccharomyces cerevisiae* on different carbon sources has also been reported (Soares Rodrigues et al. 2023). Moreover, Jia et al. (2017) and Chen et al. (2024) used proteomic techniques to study protein expression and associated metabolic pathways within microbial communities during straw fermentation. However, the mechanism based on proteomic underlying cooperative fermentation of lignocellulose biomass among two or three mixed strains remains unclear.

To improvement the nutritional value of *C. korshinskii* and further understand the differences between single and cocktail strain fermentation, the suitable strains were screened, and the true protein (TP) content in terms of the substrate composition and nitrogen conversion rates were optimized and evaluated. A comprehensive analysis of the differential protein enrichment pathways of *A. oryzae*, *S. cerevisiae* and their co-culture was carried out using an Astral-DIA proteomics strategy. The study proposes that the increased protein yield in the *A. oryzae* and *S. cerevisiae* co-culture stems from a metabolic cooperation: *A. oryzae* degrades *C. korshinskii* lignocellulose, thereby providing energy for *S. cerevisiae*, while concurrent enhancement of antioxidation and sulfur relay pathways stabilizes the fermentation process.

## Materials and methods

### Material Preparation and microorganism propagation

*Caragana korshinskii* Kom. was sampled from Ningxia, China, and stored at room temperature. It was then crushed and passed through a 1 mm sieve. *Trichoderma reesei* CGMCC-3.3711, *Trichoderma koningii* CGMCC-3.11416, and *Candida utilis* CGMCC-2.1012 used in this study were obtained from the China General Microbiological Culture Collection Center. *A. oryzae* (accession number: KU320673), *S. cerevisiae* (accession number: KM005256), *Bacillus subtilis* (KU239980), *Aspergillus niger* (accession number: SAMN46823768), and *Eurotium cristatum* (accession number: SAMN46823767) were isolated from total mixed rations silage or soil under the woody plants. Moreover, the isolated *Aspergillus*, *Eurotium*, and *Bacillus* can secrete cellulase using sodium carboxymethyl Congo red screening medium method and cellulase activities analysis according to Chen et al. (2024); Zuo et al. (2018b) (Table S1). Fungi and yeasts were maintained on potato dextrose agar slants, whereas *Bacillus* was grown on nutrient broth slants. All microbes were activated and organism suspensions were prepared according to the method described by Zuo et al. (2018a)

### Single and mixed strains solid-state fermentation

Solid-state fermentation was carried out in 250 mL conical flasks. To promote mycelial growth and microbial proliferation, the initial fermentation substrate was composed of 8 g of *C. korshinskii*, 1 g of bran, 1 g of cornmeal, 0.3 g of urea, 0.3 g of (NH_4_)_2_SO_4_, and 1 mL of salt nutrient solution with slight modification which contained the following chemicals (%): KH_2_PO_4_, 2.0; MgSO_4_⋅7 H_2_O, 0.3; CaCl_2_, 0.3; FeSO_4_⋅7 H_2_O, 0.005; MnSO_4_, 0.0016; ZnSO_4_, 0.0014; CoCl_2_, 0.002. The moisture content was adjusted to 60% with deionized water, mixed, and sterilized at 121 °C for 20 min. After all flasks cooled to room temperature, for single strain fermentation, the substrates were inoculated separately with fungal spore suspensions (1 × 10^7^ spores/g) of *T. reesei*, *T. koningii*, *A. oryzae*, *(A) niger*, and *E. cristatum*; yeast cell suspension (1 × 10^8^ cells/g) of *S. cerevisiae* and *C. utilis*; and bacterial cell suspension (1 × 10^8^ cells/g) of *(B) subtilis*. For co-culture fermentations, the fungal spore suspension was mixed with an equal volume of either the yeast or bacterial cell suspension. The strains were inoculated in an ultra-clean bench, mixed uniformly with a sterile glass rod, and then sealed with a sealing film. All experiments were conducted in a temperature-controlled incubator with a relative humidity maintained at 80%.After fermentation, the substrates were air dried at 65 °C for 48 h and pulverized to pass through a 1 mm sieve for crude protein (CP) and TP content determinations. The CP content was quantified using the Kjeldahl method. The TP content was estimated using samples precipitated with trichloroacetic acid and measured according to the Kjeldahl method (Licitra et al. 1996). Three replicates were performed for each treatment group.

### Response surface optimization

#### Plackett–Burman (PB) test

Five medium composition factors, namely bran (X_1_), cornmeal (X_2_), urea (X_3_), (NH_4_)_2_SO_4_ (X_4_), and nutrient salts (X_5_), were designed to explore the TP content of *C. korshinskii* fermentation products and determine the factors that have a greater effect on TP content. The PB test of the Design Expert software, as shown in Table S2, was used for a total of 12 sets of tests.

#### Nitrogen conversion efficiency

According to the results of analysis of the variance of the PB test (Table 1), bran and cornmeal had a significant effect on the TP content (*P* < 0.01), whereas urea and (NH_4_)_2_SO_4_ had no significant effect on the TP content (*P* > 0.01); however, it was ever reported that the inorganic nitrogen plays a key role in protein enrichment by microorganisms (Niu et al. 2025; Ren et al., 2025). Therefore, the effects of different nitrogen sources with their varying addition levels on the CP, TP content, and nitrogen conversion efficiency were further studied. Urea, (NH_4_)_2_SO_4_, and KNO_3_ were selected as different nitrogen sources and were added 0.47%, 0.94% and 1.42% of the nitrogen content of the dry matter (DM) content of the substrate, respectively. The NH_4_^+^-N and NO_3_^−^-N contents of samples after fermentation were extracted with 1 M KCl in a 1:5 (w/v) ratio of substrates to solution and measured using a continuous flow analyzer (SKALAR, Breda, Netherlands) (Zuo et al. 2022).

**Table 1:** PB test analysis of variance table

Source of error	Sum of squares	Degree of freedom	Mean square	F-value	*P*-value
Model	20.79	5	4.16	11.69	< 0.01
Bran, X_1_	10.08	1	10.08	28.37	< 0.01
Cornmeal, X_2_	10.30	1	10.30	28.99	< 0.01
Urea, X_3_	0.36	1	0.36	1.01	0.35
(NH_4_)_2_SO_4_, X_4_	0.03	1	0.03	0.07	0.80
Nutrient salts, X_5_	0.01	1	0.01	0.03	0.86
Residual	2.13	6	0.35		
Cor Total	22.92	11		*R* ^*2*^	0.907
Standard deviation	0.60			Adjusted *R*^*2*^	0.829
Mean	8.29			Predicted *R*^*2*^	0.628
C.V.%	7.19			Adeq Precision	9.72

#### Central composite design (CCD)

A three-factor, three-level CCD experiment was conducted according to the results of the PB test and different levels of nitrogen source addition test. The variables were defined as bran (X_1_), cornmeal (X_2_), and (NH_4_)_2_SO_4_ (X_3_). The center point values were 2.0 g of bran, 3.0 g of cornmeal, and 0.45 g of (NH_4_)_2_SO_4_. A total of 20 sets of experiments were conducted using Design Expert software, as shown in Table S3.

### Proteomics analysis

#### Protein extraction, quality test and trypsin treatment

According to the TP content of single and mixed strains fermentation. The samples from only sterilized substrates (CK), *A. oryzae* fermentation substrates (Aor), *S. cerevisiae* fermentation substrates (Sce) and mixed *A. oryzae* and *S. cerevisiae* fermentation substrates (MixF) were ground into powder under low temperature and lysed with an appropriate amount of SDT (4% (w/v) SDS, 100 mM Tris/HCl, pH 7.6) to extract the proteins. Briefly, the solution was sonicated on ice for 5 min, followed by centrifugation at 12,000*g* for 15 min at 4 °C. The supernatant was heated at 95 °C for 8–15 min, followed by an ice bath for 2 min. An adequate amount of iodoacetamide solution was added and incubated in the dark for 1 h. Four volumes of pre-chilled acetone (− 20 °C) were then added, and the sample was incubated at − 20 °C for at least 30 min to precipitate the proteins. After incubation, the samples were centrifuged, and the precipitate was collected, washed twice with cold acetone, air-dried, and represented the total protein. Finally, an appropriate amount of dissolved buffer was added to completely dissolve the protein pellet. The concentration of total proteins was then quantified using the Bradford protein quantitative kit according to the instructions. 20 µg of the protein sample was loaded to 12% SDS-PAGE gel electrophoresis, the gel was stained by coomassie brilliant blue R-250 and decolored until the bands were visualized clearly. An appropriate amount of protein from each sample was trypsinized according to a filter-aided proteome preparation protocol. The peptides were then desalted by using C18 Cartridges. The eluents of each sample were collected and lyophilized. Each treatment samples were carried out in triplicate.

#### LC–MS/MS analysis-DIA mode

The treated samples were separated using Ultra high-performance liquid chromatography (UHPLC). Buffer A was 0.1% formic acid aqueous solution and buffer B was 0.1% formic acid acetonitrile aqueous solution (80.0% acetonitrile). The lyophilized powder was dissolved using 10 µL buffer A solution, centrifuged at 14,000*g* for 20 min at 4 °C, and 200 ng of the supernatant sample was injected into the UHPLC. The samples were separated using a C18 analytical column (ES906 column, 15 cm, ID 150 μm, 2 μm; Thermo Fisher scientific Inc., Waltham, MA, USA) and analyzed using a thermo orbitrap astral mass spectrometer after separation, an Easy-spray (ESI) ion source was used, the ion spray voltage was set to 2.0 kV, the ion transfer tube temperature was set to 290 °C, and the mass spectrum was in a data independent acquisition mode, with a full first-stage mass spectrometry scanning range of m/z 380–980. The primary MS resolution was set to 240,000 (200 m/z), AGC was set to 500%, the parent ion window size was set to 2-Th, the number of DIA windows was 300, the NCE was set to 25%, the secondary m/z acquisition range was from 150 to 2000, the sub-ion resolution Astral was set to 80,000, and the maximal injection time was 3ms.

#### LC–MS/MS data analysis

The raw files were analyzed by searching against the UniProt protein database using the DIA-NN library search software. This process was performed by Novogene Co. Ltd (Tianjin, China). The mass deviation of precursor ions and fragment ions is automatically detected and corrected. The maximum number of missed cleavage sites was set to 2, with the peptide length set to 7–25 amino acids for residues and cysteine alkylation set as fixed modification. To improve the quality of analysis results, the DIA-NN software further filters the search results, retaining only peptides with a Global.Q.Value < 0.01 and proteins with a PG.Q.Value < 0.01. A protein with a fold change (FC) greater than or less than a certain value (FC) was defined as a differentially expressed protein (DEPs).

Gene Ontology (GO) and InterPro (IPR) functional analysis were conducted using the interproscan program against the non-redundant protein database (including Pfam, PRINTS, ProDom, SMART, ProSite, PANTHER) (Huang et al. 2009), and the databases of COG (Clusters of Orthologous Groups) and KEGG (Kyoto Encyclopedia of Genes and Genomes) were used to analyze the protein family and pathway. DEPs were used for Volcanic map analysis, cluster heat map analysis and enrichment analysis of GO, IPR and KEGG (Franceschini et al. 2012).

### Amino acid (AA) content determination

Amino acid content was determined using acid hydrolysis, derivatization, and HPLC quantification according to the methods described by Frias et al. (2008) and Zhu et al. (2016). In brief, fermented and unfermented samples were hydrolyzed with 6 M HCl for 24 h at 110 °C in a vacuum sealed vial. The hydrolysate was dried with N_2_ and redissolved in water. For AA derivatization, phenyl isothiocyanate and triethylamine (99%, Sigma-Aldrich, St. Louis, MO, USA) were used. Amino acid profiles were quantified using an HPLC system (Shimadzu Corporation, Kyoto, Japan) equipped with a UV detector (SPD-20AV). The sample (20 µL) was injected into a C18 column (5 μm, 4.6 × 250 mm) at 30 °C. The linear-gradient system with buffer A (0.1 M NaAc: acetonitrile 97: 3, v/v, pH 6.5) and buffer B ( acetonitrile: water 4: 1, v/v) at pH 6.5 enabled the separation of the AAs. The analytes were quantified using a UV detector at 254 nm.

### In vitro digestibility

Samples were analyzed in triplicate using an in vitro digestion model for ruminants to evaluate the in vitro digestibility of DM, OM, CP and TP from the SSF process and raw materials (Tilley 1963). Air-dried unfermented and mixed strains of *A. oryzae* and *S. cerevisiae* fermented samples (0.5 g) were weighed into nylon filter bags (Ankom F57; ANKOM Technology, Fairport, NY, USA) and sealed. Rumen cannulated steers fed a basal diet were used in an in-situ study to supply inoculum for the in vitro digestibility study (Zheng et al. 2022). Rumen fluid was collected from steers fed a corn silage-based diet and mixed with a buffer solution under anaerobic conditions. The filter bags containing the samples were then incubated in 40 mL of buffered rumen fluid at 39 °C for 48 h. After incubation, the filter bags were removed from the tubes and washed with distilled water. Standard procedures were used to calculate the digestibility of DM (DMD), OM(OMD), CP (CPD), and TP (TPD).

### Data analysis

Data from three independent trials (*n* = 3) were subjected to statistical analyses. Analysis of variance (ANOVA) was performed using the SPSS software (version 25.0; SPSS Inc., Chicago, IL, USA). LSD and Duncan’s tests were applied when ANOVA revealed significant differences (*P* < 0.05) between samples.

## Results

### Screen strains

**Fig. 1:**
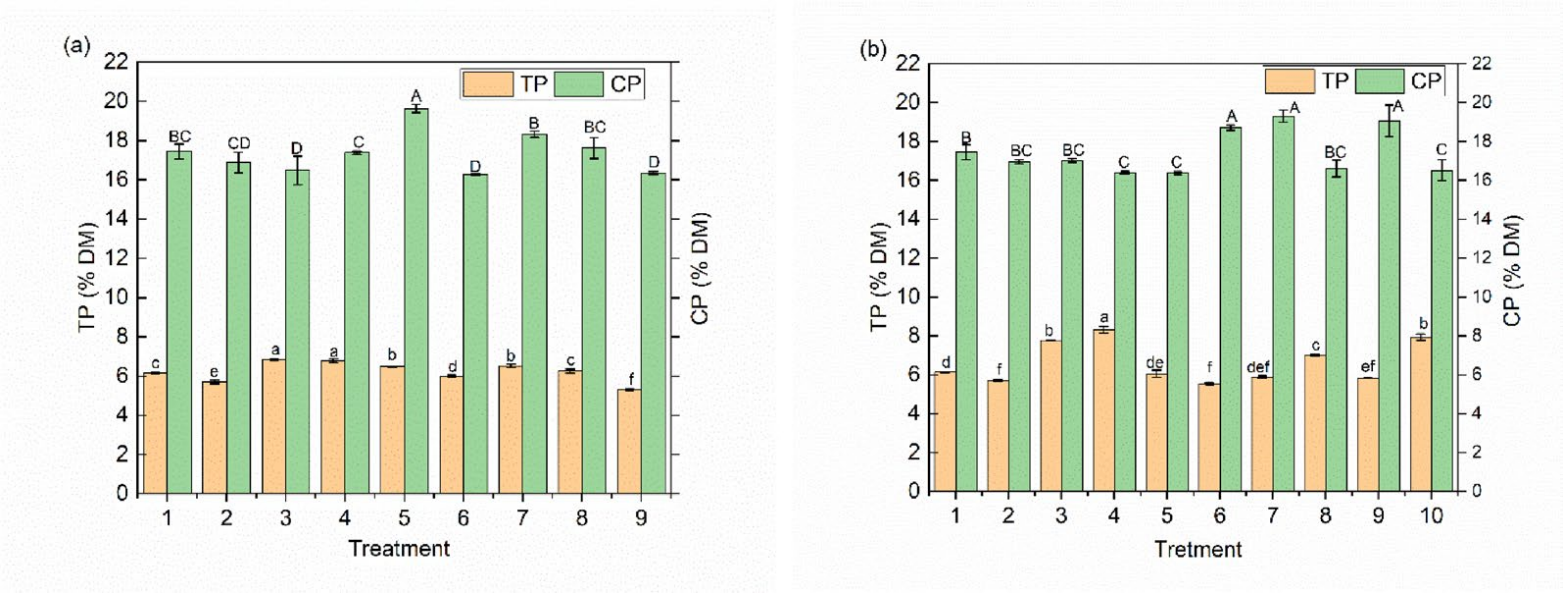
Effect of solid-state fermentation on TP and CP content of the fermented substrates. **a** Single-strain SSF (1, *control*; 2, *A. niger*; 3, *(A) oryzae*; 4, *E. cristatum*; 5, *T. reesei*; 6, *T. koningii*; 7, *S. cerevisiae*; 8, *C. utilis*; 9, *(B) subtilis*). **b** Mixed-strains SSF (1, control; 2, *E. cristatum* + *A. oryzae*; 3, *E. cristatum* + *S. cerevisiae*; 4, *A. oryzae* + *S. cerevisiae*; 5, *A. oryzae* + *B. subtilis*; 6, *E. cristatum* + *B. subtilis*; 7, *S. cerevisiae* + *B. subtilis*; 8, *A. oryzae* + *S. cerevisiae* + *B. subtilis*; 9, *E. cristatum* + *S. cerevisiae* + *B. subtilis*; 10, *E. cristatum* + *A. oryzae* + *S. cerevisiae* + *B. subtilis*). Different *letters* (*a-f*; A-D) indicate significant difference (*P* < 0.05) according to LSD and Duncan’s test TP, true protein; CP, crude protein; SSF, solid state fermentation

The isolated *Aspergillus*, *Eurotium*, and *Bacillus* can secrete cellulase using sodium carboxymethyl congo red screening medium method (D/d > 1), with their cellulase activities decreasing in the following order: *Aspergillus* > *Bacillus* > *Eurotium* > *T. reesei* (Table S1). The TP and CP contents of the substrates after single and mixed strains fermentation with various microbes are shown in Fig. 1a, b. The substrates inoculated with *A. oryzae* had the highest (*P* < 0.01) TP content (6.85%), followed by *E. cristatum* (6.78%), *S. cerevisiae* (6.55%) and *T. reesei* (6.49%). Considering the more safety and potential synergy, *(A) oryzae*, *E. cristatum*, *S. cerevisiae* and *(B) subtilis* were further selected for the mixed strains fermentation. Although the substrates fermented with *A. oryzae* and *S. cerevisiae* had significantly lower (*P* < 0.01) CP contents, they had significantly higher (*P* < 0.01) TP contents than the substrates fermented with other microbial combinations, with the highest (*P* < 0.01) TP content of 8.32% in the fermentation product.

### Optimization

#### Effects of optimization of medium composition by PB design on TP content

The ANOVA results of PB are presented in Table 1. The model’s *P*-value of less than 0.01 indicated that it was statistically significant (*P* < 0.01). The *R*^*2*^ and adjusted *R*^2^ were 0.907 and 0.829, respectively, which were close to 1, indicating a good correlation. Analysis of the comparative *P* values showed that the magnitude of the effect on TP content was in the order of cornmeal > bran > urea > salt nutrient solution >(NH_4_)_2_SO_4_. The effects of bran and cornmeal were significant (*P* < 0.01), whereas those of urea and (NH_4_)_2_SO_4_ were not significant (*P* > 0.05). The regression equation was as follows:

1 $$ \begin{aligned} R_{1} & = 8.24 + 0.873X_{1} + 0.883X_{2} \\ & \quad - 0.129X_{3} - 0.0025X_{4} + 0.0125X_{5} \\ \end{aligned} $$

where Y was the yield of TP, and X_1_, X_2_, X_3_, X_4_, and X_5_ were the coded variables for wheat bran, cornmeal, urea, (NH_4_)_2_SO_4_ and salt nutrient solution, respectively.

#### Nitrogen utilization by mixed strains fermentation

Experiments were conducted according to further clarify the effects of different types of nitrogen sources and addition levels on protein production by *A. oryzae* and *S. cerevisiae* combined with the fermentation of the substrates (Table 2). The results showed that, under the same conditions of incubation, the addition of low concentrations of (NH_4_)_2_SO_4_ resulted in the highest (*P* < 0.01) conversion rate of nitrogen source into protein, which reached 69%. The content of NH_4_^+^-N or NO_3_^−^-N of the substrate after fermentation increased with increasing amounts of different nitrogen sources. The difference between the theoretical unutilized nitrogen and the actual nitrogen content measured after the fermentation in the urea addition treatment group was the largest (*P* < 0.01).

**Table 2:** Nitrogen addition affects conversion efficiency in *A. oryzae* and *S. cerevisiae* fermentation of *C. korshinskii*

Treatment	Urea (g)	(NH_4_)_2_SO_4_ (g)	KNO_3_ (g)	CP (%)	TP (%)	Conversion rate%	F-NH_4_^+^ (mg/L)	F-NO_3_^−^ (mg/L)	Gap-*N* (g)
Control	0.00	0.00	0.00	8.04	6.88	0	0.69	0.11	0.00
Urea1	0.10	0.00	0.00	13.09	9.85	41	1.00	0.12	1.51
Urea2	0.20	0.00	0.00	13.37	9.34	14	4.60	0.11	2.63
Urea3	0.30	0.00	0.00	14.81	8.76	17	7.35	0.12	3.79
(NH_4_)_2_SO_4_ 1	0.00	0.22	0.00	13.04	10.39	69	0.80	0.10	0.12
(NH_4_)_2_SO_4_ 2	0.00	0.45	0.00	15.52	11.11	58	3.13	0.11	0.25
(NH_4_)_2_SO_4_ 3	0.00	0.67	0.00	17.43	10.74	37	6.81	0.12	0.36
KNO_3_ 1	0.00	0.00	0.33	12.12	8.88	21	1.64	0.67	0.86
KNO_3_ 2	0.00	0.00	0.67	14.16	8.56	12	1.61	4.05	1.29
KNO_3_ 3	0.00	0.00	1.01	15.68	8.39	9.6	1.61	9.39	0.47

F-NH_4_^+^, ammonium nitrogen in the substrates after fermentation; F-NO_3_^−^, nitrate nitrogen content in the substrates after fermentation; Gap-N, based on 1000 g of substrate, N difference between the theoretically ungenerated N and the actual measured residue N (fermentation sample); Control, the substrate without nitrogen addition and only sterilized

#### Effect of CCD experiment on the TP content

**Fig. 2:**
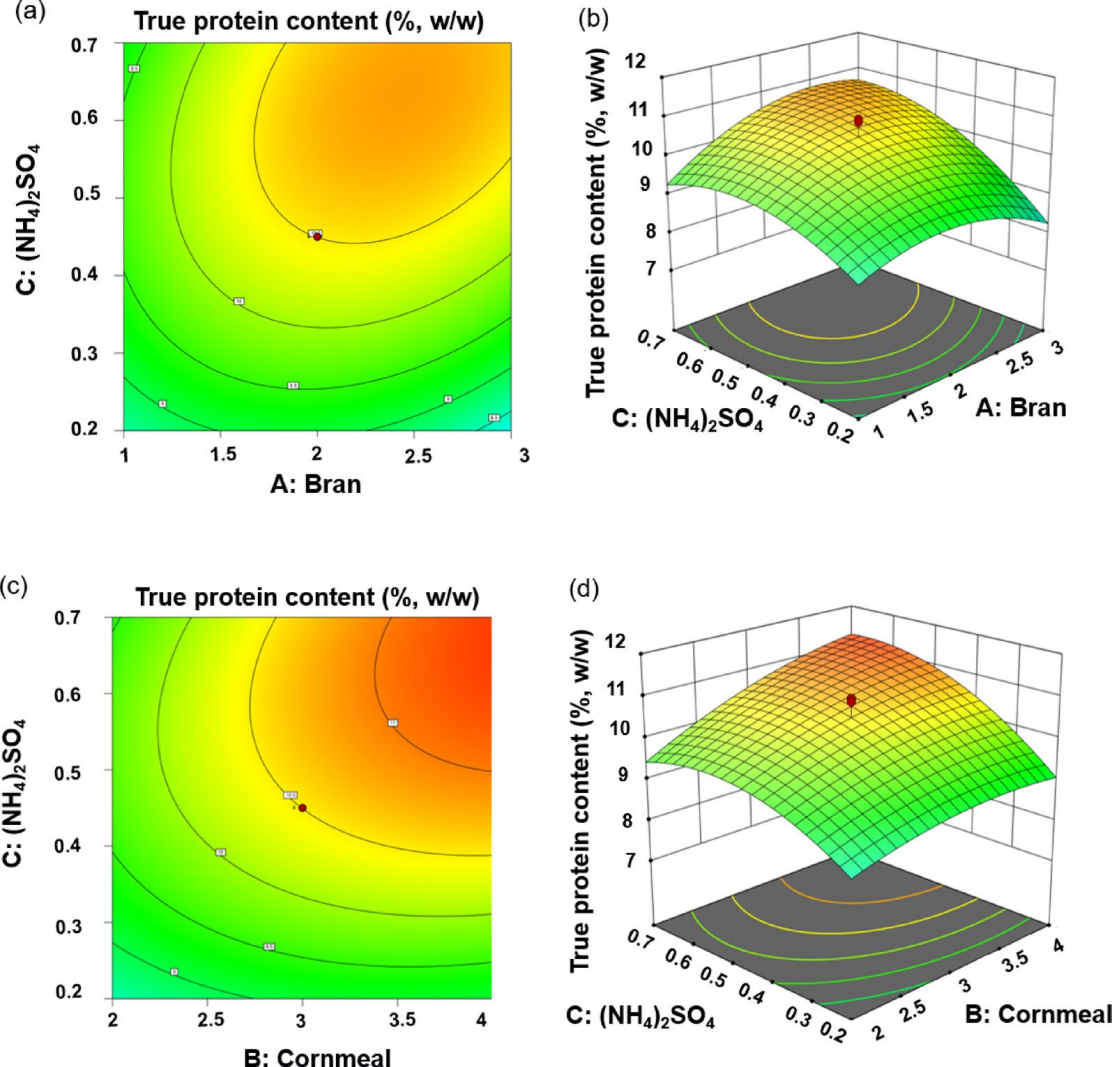
Effects of (NH_4_)_2_SO_4_ & bran (**a**, **b**) and (NH_4_)_2_SO_4_ & cornmeal (**c**, **d**) on TP yield. **a** and **c**, contour plots; **b** and **d**, response surface plots

The ANOVA results of the CCD are presented in Table S4. ANOVA analysis showed that the F value of the model was 21.9, with a *P* value less than 0.01, and the model’s *R*^*2*^ and Adj *R*^2^ were 0.952 and 0.908, which were close to 1. There was a significant interaction between (NH_4_)_2_SO_4_ and bran, and (NH_4_)_2_SO_4_ and cornmeal content, which affected TP content (Fig. 2). According to the analysis using Design Expert 13 software, the following compositions can be recommended as the practical optimum: wheat bran content of 20%, corn powder content of 30%, and (NH_4_)_2_SO_4_ content of 5%. A multiple regression analysis of the experimental data led to the following regression equation:

2 $$ \begin{aligned} Y & = 10.5 + 0.267X_{1} + 0.588X_{2} + 0.774X_{3} \\ & \quad + 0.254X_{1} X_{2} + 0.439X_{1} X_{3} + 0.329X_{2} X_{3} \\ & \quad - 0.658X_{1}^{2} - 0.278X_{2}^{2} - 0.644X_{3}^{2} \\ \end{aligned} $$

where Y was the TP yield, and X_1_, X_2_, and X_3_ were the coded variables for wheat bran, cornmeal, and (NH_4_)_2_SO_4_, respectively.

### Proteomic analysis

**Fig. 3:**
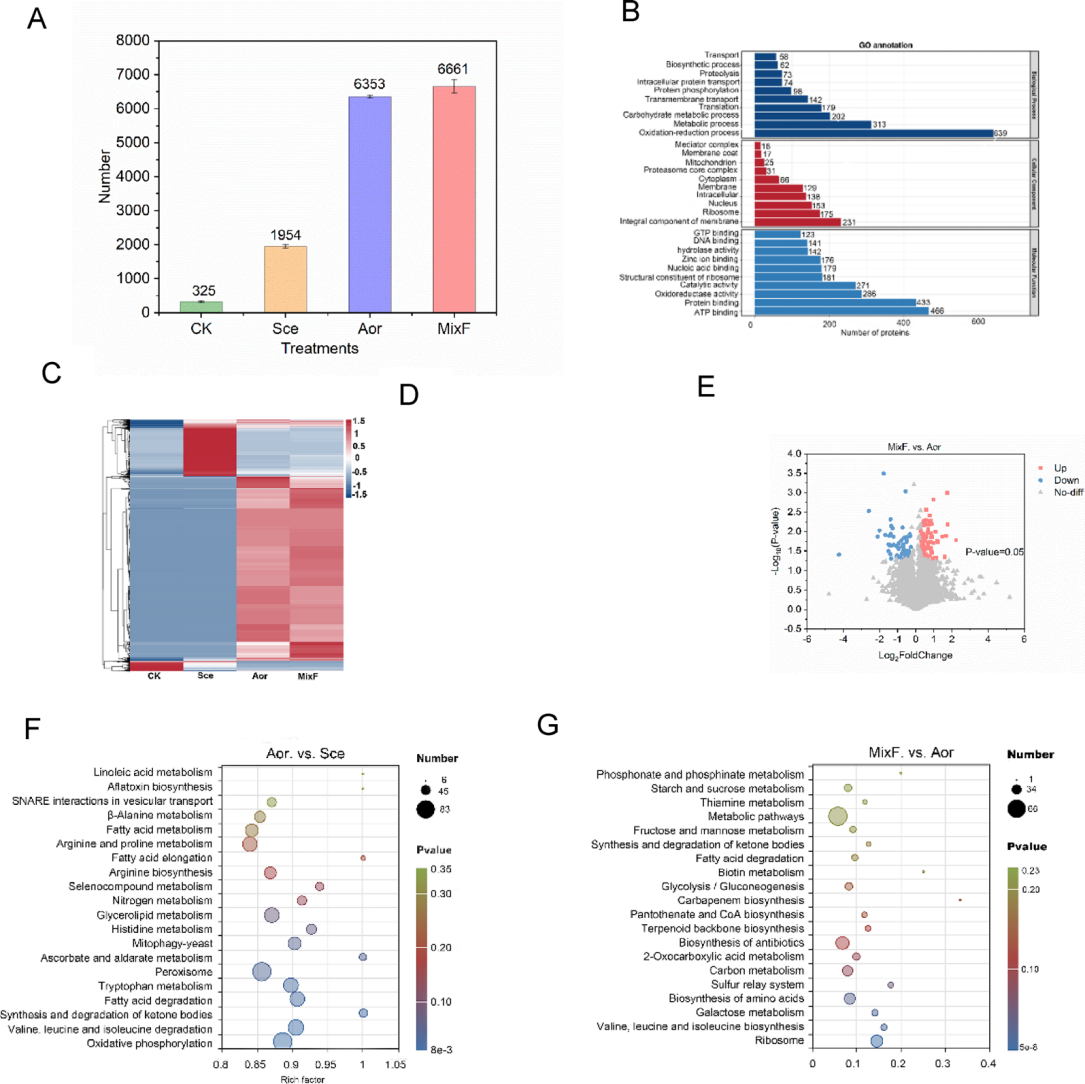
Proteomics analysis. Protein identification overview chart (**A**), GO annotation result bar chart (**B**) and the differential protein clustering heatmap (**C**) of the proteome samples of only sterilized substrates (CK), *Saccharomyces cerevisiae* (Sce), *Aspergillus oryzae* (Aor), and the mixed *S. cerevisiae* and *A. oryzae* fermented substrates (MixF); differential protein volcano plot and KEGG enriched bubble chart between Aor and Sce (**D** and **F**), MixF and Aor (**E** and **G**)

A total of 8011 proteins and 69,892 peptides were identified across the four treatment groups (Excel sheet 1). Figure 3A shows that the CK group had the lowest number of proteins (325 ± 2), significantly lower than all microbial treatment groups (*P* < 0.01). The proteins in Aor group (6353 ± 24) were significantly higher than the Sce group (1954 ± 32) (*P* < 0.01), while the MixF group had the highest count (6661 ± 190) (*P* < 0.01). Figure 3B shows the GO functional annotation of total proteins. These proteins were primarily involved in biological processes such as oxidation-reduction process (639 proteins), metabolic processes (313 proteins), and carbohydrate metabolism (202 proteins). Regarding cellular components, integral membrane proteins (231 proteins) and ribosomes (175 proteins) were the most abundant. For molecular functions, ATP binding (466 proteins), protein binding (433 proteins), oxidoreductase activity (286 proteins), and catalytic activity (271 proteins) were predominant. Both PCA and Venn diagrams further demonstrated significant differences in proteins among the four treatment groups (Fig. S1a, b ).

The comparative heatmap of DEPs among the four treatment groups (Fig. 3C) revealed that the numbers of upregulated proteins in both MixF and Aor groups were significantly higher than that in Sce and CK groups, and fewer than 300 different proteins in the MixF and Aor groups (Table S5, Excel sheet 2-4). Figure 3D displays the volcano plot of the Aor versus Sce comparative, which identified 129 significantly upregulated, 292 downregulated proteins, and 254 non-differential proteins. However, our protein identification analysis (Table S5, Excel Sheet 2) revealed that 4,875 proteins were uniquely detected in the Aor group, while 845 were exclusive to the Sce group. Similarly, supplementary Fig. S1c shows that the the MixF versus Sce comparison resulted in 106 upregulated and 800 downregulated DEPs. Complementing this, Table S5, Excel Sheet 3 shows that 4,543 proteins were unique to the MixF group, compared to only 309 unique to the Sce group. Figure 3E presents the volcano plot for the MixF versus Aor group, which identified 85 significantly upregulated proteins (including protein P29453 with log2FC = 2.23, indicating a ~ 4.7-fold increase) and 67 downregulated proteins. Furthermore, the identification analysis revealed 127 proteins unique to the MixF group, and only 12 unique to the Aor group (Table S5, Excel Sheet 4).

Figure 3F, G and Fig. S1d display the KEGG enrichment bubble plots of DEPs in the Aor versus Sce, MixF versus Aor, and MixF versus Sce groups, respectively. The Aor versus Sce analysis revealed that these proteins were primarily enriched in the following pathways: oxidative phosphorylation, valine, leucine and isoleucine degradation, ketone body synthesis and degradation, fatty acid degradation, tryptophan metabolism, and peroxisome-related processes. The MixF versus Aor analysis demonstrated significant enrichment of proteins in the metabolic pathways of ribosome biosynthesis, valine/leucine/isoleucine biosynthesis, galactose metabolism, amino acid biosynthesis, and sulfur relay systems. The MixF versus Sce comparison showed significant enrichment of proteins in the peroxisome pathway (Fig. S1d), and the relative abundance of protein A0AAN4YNS0 (related to the peroxisome) in the MixF group was higher than that in the Sce group (Supplementary Excel sheet5). Supplementary Fig. S1e and f display chord diagrams of Go-enriched and KEGG-enriched DEPs in the MixF versus Aor group. As shown in Supplementary Excel Sheet5 and the chord diagrams, the key enriched proteins include: ribosomal protein A0A1S9DZ50, which was involved in protein synthesis as a component of the ribosome; A0A1S9DR51, associated with valine, leucine, and isoleucine biosynthesis; Chorismate synthase A0A1S9DNX6; and proteins linked to the synthesis of specific amino acids: Q2TX96 (tryptophan), C8VT45 (arginine), and A0A1S9DX19 (proline); as well as sulfur transfer-related protein A0A1S9DI65.

### AA composition analysis

**Fig. 4:**
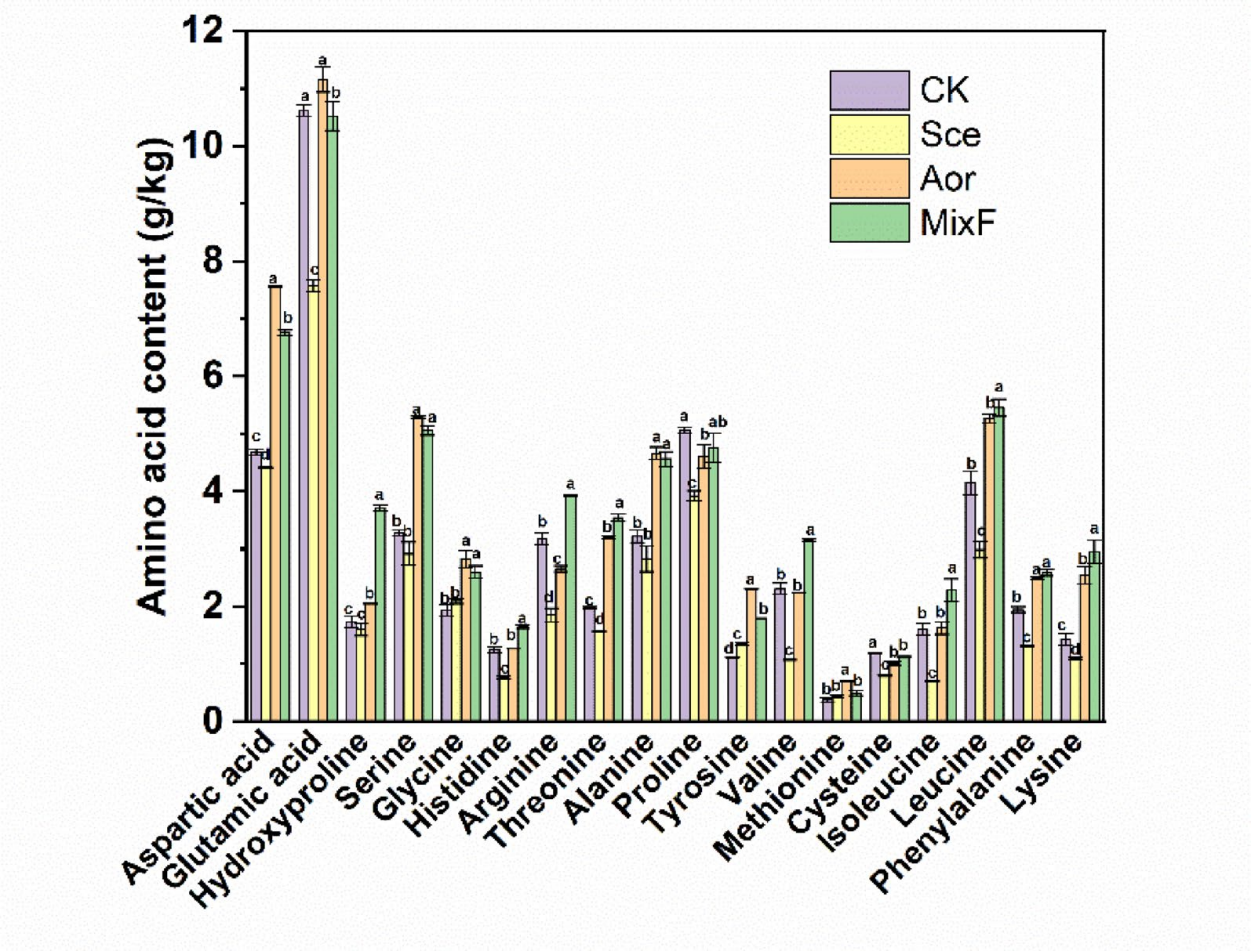
Changes in amino acid content in the *C. korshinskii*-based substrate before and after fermentation. Different lowercase letters for the same amino acid indicate significant differences between different treatment groups (*P* < 0.05). CK, substrate only sterilized; Sce, substrate fermented with *S. cerevisiae*; Aor, substrate fermented with *A. oryzae*; MixF, substrate fermented with mixed *A. oryzae* and *S. cerevisiae*

The AA compositions before and after fermentation are shown in Fig. 4. After fermentation with *S. cerevisiae*, only the tyrosine content slightly increased compared to the CK, however, in the Aor group, the tyrosine content increased by 2-fold. Moreover, the contents of aspartic acid, serine, threonine, methionine, lysine also increased significantly in the Aor group. The contents of aspartic acid, hyroxyproline, serine, glycine, histidine, arginine, threonine, alanine, tyrosine, valine, methionine, isoleucine, leucine, phenylalanine, lysine in the MixF group increased by 44.4%, 114%, 53.8%, 33.8%, 31.3%, 23.5%, 79.0%, 41.4%, 61.1%, 36.9%, 29.3%, 42.3%, 31.6%, 33.1% and 106%, respectively.

### In vitro digestibility analysis

Table 3 shows DMD, OMD, CPD and TPD of the substrates before and after fermentation. The in vitro DMD and CPD did not differ significantly before and after fermentation. However, OMD and TPD was higher in the MixF than in the unfermented substrate, and TPD increased by more than 17%.

**Table 3:** In vitro digestibility of nutrients in the unfermented and fermented substrates for beef cattle

Nutrients digestibility (%)				
	DMD	OMD	CPD	TPD
Substrates				
CK	47.45 ± 0.54^A^	46.31 ± 0.76^B^	74.37 ± 1.72	50.44 ± 2.88^B^
MixF	44.79 ± 1.40^B^	48.74 ± 1.14^A^	76.54 ± 1.44	67.24 ± 3.62^A^

DMD, OMD, CPD, TPD, digestibility of dry matter, organic matter, crude protein and true protein. Treatments: CK, non-fermented mixture of *C. korshinskii*; MixF, mixture of *C. korshinskii* fermented with *A. oryzae* and *S. cerevisiae.* The data in the table is presented as mean ± standard deviation. Values with different superscripts within a column (A-B) are significantly different (*P* < 0.05).

## Discussion

### Strain and medium compositions influencing TP content

The substrates inoculated with *A. oryzae*, *E. cristatum*, *S. cerevisiae* and *T. reesei* had higher TP contents than the other strains (Fig. 1), suggesting that these fungi may be useful for increasing the TP content in *C. korshinskii*. It was well-established that *T. reesei* possesses a remarkable ability to degrade cellulose, relying on its secreted cellulases. However, in our study, its cellulase activity was considerably lower than that of *(A) oryzae*, *E. cristatum* and *(B) subtilis* (Table S1). This is probably because *T. reesei* does not preferentially produce cellulase on the substrate with *(C) korshinskii* as the main component. Furthermore, although the lysozyme preparation produced by *T. reesei* DSM 32,338 was authorized in the EU as a feed additive for laying hens in January 2025. Adequate studies on the safety and efficacy of *T. reesei* as a feed additive are still lacking. *E. cristatum* is valued in Fuzhuan brick tea for forming the characteristic “golden flowers” and producing compounds that enhance flavor and aid digestion. It is also recognized as a regulated, safe food fungus (Zou et al. 2019). Nevertheless, compared to *A. oryzae*, it exhibits slower growth and is more susceptible to microbial contamination. Therefore, selecting *A. oryzae* and *S. cerevisiae* for subsequent experiments appears to be more sensible choice. Moreover, the substrates fermented with *A. oryzae* and *S. cerevisiae* had significantly higher TP contents than those fermented with other combinations.

Many studies have focused only on the effects of single strains of *A. oryzae* or *S. cerevisiae* on the protein content after fermentation (Bátori et al. 2015; Mao et al. 2013). However, studies investigating the synergistic fermentation of the two strains are limited. Thongkratok et al. (2010) only measured the increase in the CP content of cassava pulp after fermentation with *A. oryzae* and *S. cerevisiae*; however, they did not analyze the change in TP content, which can better reflect the inorganic nitrogen conversion rate by microorganisms. Moreover, the synergistic of *A. oryzae* and *S. cerevisiae* fermentation of the substrates was not clear. We speculate that *A. oryzae* has greater cellulase activity, which enables cellulose degradation during *C. korshinskii* fermentation to produce an available carbon source. Second, sugars released from cellulose have been demonstrated significant inhibitory effects on the activity of both β-glucosidase and cellulase mixtures (Xiao et al. 2004); S. *cerevisiae* can consume these sugars, which relieves the inhibition of the hydrolases and allowing continued hydrolysis, thereby supporting the growth of both strains. Further research is required to confirm these two factors are responsible for the observed synergistic results.

When many factors and interactions affect the desired responses, the response surface method is an effective tool for optimizing the process. It uses an experimental design, such as PB and CCD, to fit a model using the least-squares technique. If the proposed model is adequate, as revealed by the diagnostic tests provided by the ANOVA and residual plots, contour plots can be efficiently used to study the response surface and identify the optimum conditions (Zuo et al. 2018a). The PB model had high F-value (*P* < 0.05) and Adeq Precision (Table 1), which suggested that the model with a good fit was precise and reliable. The *P*-value of wheat bran and cornmeal were less than 0.01 indicating that they were suitable for the growth of the screened strains. Although the *P*-value of (NH_4_)_2_SO_4_ and urea were greater than 0.05, inorganic nitrogen can be converted into organic nitrogen by microorganisms (Zhao et al. 2022), therefore, further optimization of nitrogen addition is necessary. Our preliminary experiments with urea additions of 0, 0.2, 0.6, 0.8, and 1.0 g revealed that microbial growth was severely inhibited when urea exceeded 0.6 g, demonstrating the growth-suppressing effect of excessive nitrogen. Based on this finding and the reference level of 0.84% DM nitrogen addition reported by Ren et al. (2025), we designed experiments with addition amounts both lower and higher than this value.

The low concentration of (NH_4_)_2_SO_4_ resulted in the highest conversion rate of the nitrogen source into protein (Table 2), which may be because (NH_4_)_2_SO_4_ is easier than urea and KNO_3_ absorbed by *A. oryzae* and *S. cerevisiae*. Compared to KNO_3_, (NH_4_)_2_SO_4_ may promote the synthesis of AA by microorganisms as a nitrogen source (Zhao et al. 2022). In addition, the addition of a low concentration of (NH_4_)_2_SO_4_ can increase the conversion rate of inorganic nitrogen by microorganisms. Moreover, based on 1000 g of substrate, the nitrogen difference between the theoretically un-utilized nitrogen and the actual measured nitrogen of residue was the lowest in the (NH_4_)_2_SO_4_ group and highest in the urea group. This observation indicated that (NH_4_)_2_SO_4_ was utilized most efficiently. On the contrary, some nitrogen oxide gases were probably generated and volatilized, resulting in nitrogen loss in the urea addition group.

The CCD model had a high *F* value (*P* < 0.05), a non-significant lack of fit, and a large determination coefficient *R*^2^, suggesting that the model with a good fit was precise and reliable (Table S4). Three-dimensional (3D) response surfaces provided a better way to visualize the interaction of independent variables with response values, as shown in Fig. 2. It was found that (NH_4_)_2_SO_4_ had the greatest impact on the TP yield, with a noticeable increase in yield, followed by a slight decrease as the (NH_4_)_2_SO_4_ content increased. Many fermentation experiments have been related to optimizing fermentation conditions (Liu et al. 2023; Sun et al. 2024), and there are few reports on optimizing substrate components to improve protein content. In this study, the fermentation conditions were optimized and it seems that they had no significant effect on the TP content improvement (data not shown). However, after substrate optimization, the TP content increased from 6.14% DM to 10.5% DM, suggesting that appropriate types, concentrations, and ratios of carbon and nitrogen sources can ensure efficient microbial synthesis of amino acids, thereby promoting protein accumulation (Ye et al. 2025).

### Functional and pathway enrichment analyses

Solid-state fermentation involves interactions among substrates, enzymes, and microorganisms (Chen et al. 2024). Carbon and nitrogen are the primary factors that influence the growth of microorganisms. The filamentous fungus *A. oryzae* can secrete a variety of extracellular enzymes, such as cellulases and xylanases, which break down lignocellulosic biomass into simple sugars like glucose. These sugars can be utilized by *A. oryzae* and *S. cerevisiae* and metabolized via glycolysis into pyruvate—a central metabolic intermediate (Zhang et al. 2013). Pyruvate is further processed into acetyl-CoA, entering the TCA cycle to support energy production and biosynthesis (Chen et al. 2024). Meanwhile, the TCA cycle also indirectly affects the production of amino acids such as aspartic acid, and directly influences lysine synthesis (Del Cerro et al. 2021). Previous studies have demonstrated that nitrogen sources significantly influence protein biosynthesis and substrate utilization (Ren et al. 2025; Niu et al. 2025). The intricate interplay between carbon and nitrogen metabolism is fundamental for optimizing SSF efficiency. In addition, the shift towards energy-efficient nitrogen assimilation pathways enables more effective nitrogen utilization, thereby potentially redirecting energy into elevated production of proteins and lignocellulolytic enzymes (Ren et al. 2025).

In this study, *A. oryzae* was dominant in mixed-culture fermentation, suggesting its superior protein production capacity, consistent with the significantly higher protein content observed in single-strain fermentation (Fig. 3A and C). Moreover, the proteins produced by *A. oryzae* in mixed culture were primarily involved in energy metabolism and substrate degradation (Fig. 3B). Figure 3C further demonstrated that, compared to single-strain *S. cerevisiae* fermentation, the introduction of *A. oryzae* in mixed-culture fermentation led to highly expressed proteins. Meanwhile, proteins that were downregulated (though highly expressed in the Sce group) suggested that *A. oryzae* might inhibit the peroxisome pathway of *S. cerevisiae* during mixed-culture fermentation (Excel Sheets 6 and 7). KEGG pathway analysis revealed upregulated oxidative phosphorylation and fatty acid degradation pathways in *A. oryzae* compared to *S. cerevisiae*, suggesting *A. oryzae* preferentially employs these more efficient ATP-generating metabolic strategies (He et al. 2019). The degradation of valine, leucine, isoleucine and tryptophan may provide carbon or nitrogen sources for *A. oryzae* (Li et al. 2023). Moreover, the enrichment of peroxisome-related proteins in the Aor group was associated with lipid metabolism and antioxidant stress response (Orosz et al. 2017). Although it has been reported that some filamentous fungi can secrete large amounts of protein into the growth medium compared to model microorganisms such as *S. cerevisiae* (Sakuragawa et al. 2021), the detailed mechanisms of microbe-microbe and microbe-host interactions often remain unclear (Zaramela et al. 2021).

Ribosomes are the factories for protein synthesis (Cech 2000). The ribosome pathway was significantly enriched in MixF compared to Aor (23 proteins, *P* = 1 × 10^−8^), suggesting enhanced translation efficiency to meet dual-species metabolic demands. However, only one protein (A0A1S9DZ50, ribosomal protein S13) was detected in Aor, and at lower levels than in MixF. This implies that S13 deficiency in Aor may limit ribosomal activity, while its higher abundance in MixF could contribute to protein enrichment. Cukras et al. (2003) reported that modification or removal of S13 in *E. coli* disrupted a communication network critical to translocation. Therefore, further verification of A0A1S9DZ50’s role is essential to elucidate the mechanism underlying the higher protein content in MixF compared to Aor. Key enzymes for valine, leucine, and isoleucine biosynthesis, such as dihydroxy-acid dehydratase (A0A1S9DR51), were significantly upregulated. This suggests that the mixed-culture system enhances valine, leucine, isoleucine synthesis, potentially to support mycelial growth or serve as a nitrogen source for *S. cerevisiae*. This is consistent with the report by Zhou et al. (2023), who found that bacterial co-culture increased amino acid metabolism in *A. oryzae*, leading to higher protease secretion during solid-state fermentation of Jiangqu.

The significant differences in galactose metabolism (*P* < 0.05) suggest that the mixed-culture system utilizes complex carbon sources (e.g., galactose) more efficiently. This may involve synergistic interactions with hydrolytic enzymes of *A. oryzae* such as cellulase Q2TZU7, which was significantly higher in the Aor group than in other groups (Excel Sheet1). The elevated cellulase activity could facilitate the breakdown of cellulose-derived sugars, indirectly supporting galactose metabolism. Nineteen enriched proteins were associated with amino acid biosynthetic pathways, particularly the synthesis of tryptophan, arginine, and proline. This reflects nitrogen metabolic reprogramming in the *A. oryzae* and *S. cerevisiae* co-culture system. Such reprogramming could potentially enhance precursor supply for secondary metabolites (MéndezHernández et al. 2023), though further validation is needed. The enrichment of pathways related to ribosome biogenesis and amino acid metabolism observed in the mixed-culture system suggests that microbial co-existence may activate metabolic networks that are latent in monocultures, potentially through resource complementarity. In addition, the key sulfur transfer system protein A0A1S9DI65, which is associated with molybdopterin synthase catalytic subunit, is involved in the biosynthesis of cofactor (e.g., iron-sulfur cluster), and supports oxidative stress response and secondary metabolism. The *cnxH* gene, which encodes the molybdopterin synthase catalytic subunit protein A0A1S9DI65, and is highly expressed in sulfur transformation in the co-culture system, can be knocked out to further investigate its impact on microbial interactions.

Studies have shown that fermentation increases amino acid (AA) content in various foods, including *Moringa oleifera* leaf meal (Shi et al. 2021), Hong Qu (Liang et al. 2020), and other food sources (Sharma et al. 2020). This study further analyzed AA content changes before and after *A. oryzae*, *S. cerevisiae* and their mixed strains fermentation of *C. korshinskii*, to investigate how changes in amino acid content correlate with the functional expression of microbial proteins. In our study, proteins involved in the amino acid biosynthesis pathway were up-regulated in the MixF group, these findings are consistent with our AA analysis result, which showed increased relative abundance of these amino acids after fermentation (Fig. 4).

After substrate fermentation, the OM and TP digestibility were 48.7% and 67.2%, respectively (Table 3), corresponding to increases of approximately 2% and 17%. Thus, SSF promoted the OM and protein digestion of the substate mainly composed of *C. korshinskii*. Since the OM fraction excludes minerals and inorganic compounds, its organic components are more readily degraded by rumen microorganisms following SSF. Consequently, OM digestibility increased while DM digestibility decreased. Previous studies have consistently demonstrated enhanced protein digestibility in SSF products. For instance, Zuo et al. (2018a) observed a 6% improvement in CP digestibility (81.9% to 87.9%) during *A. oryzae* and *Bacillus subtilis* fermentation of sweet potato beverage residue. Similarly, Chen et al. (2024) reported a more substantial 21.8% increase in TP digestibility (45.3% to 67.1%) when fermenting corn straw with a synthetic microbiome.

It was mentation that conventional laboratory-scale spore production of *Aspergillus* sp. on slants, Petri plates, and Fernbach flasks with defined media is expensive, laborious, and not easily scalable. This creates a demand for an economical and simple mass production system. While large-scale methods using natural or inert supports have been reported (Bapat et al. 2003), an alternative approach involves adapting the traditional Qu in Chinese Baijiu or Japanese koji cultivation method to produce spores of *A. oryzae* at large scale, which can subsequently be preserved by spray drying or freeze drying for the industrial-scale application demands.

## Conclusions

The mixed culture of *A. oryzae* and *S. cerevisiae* significantly improved the TP content of *C. korshinskii*. Response surface methodology (RSM) determined the optimal substrate composition for protein enrichment: 20% bran, 30% corn powder, and 50% *C. korshinskii*. A low-level addition of (NH_4_)_2_SO_4_ proved most effective for protein enrichment. Proteomic analysis revealed up-regulation of key proteins, including ribosomal protein S13 (A0A1S9DZ50) and multiple amino acid biosynthesis-related proteins (A0A1S9DR51, A0A1S9DNX6, A0A1S9DX19), consistent with observed amino acid content changes. The sulfur transfer-related protein A0A1S9DI65, highly expressed during sulfur transformation in co-culture, represents a potential knockout target for investigating microbial interactions. This study not only provides an effective strategy for upgrading *C. korshinskii* to protein feed but also elucidates the underlying proteomic mechanisms of *A. oryzae* and *S. cerevisiae* synergy.

## Supplementary Information

Below is the link to the electronic supplementary material.

Supplementary Material 1

Supplementary Material 2

## Data Availability

All data generated or analyzed during this study are included in this published article (and its supplementary information files).
