# Supplementary material for: Valorization of Caragana korshinskii Kom. using cooperative Aspergillus oryzae and Saccharomyces cerevisiae to produce fermented feed protein
Source: Bioresour Bioprocess. 2025 Nov 5;12(1):128. doi: 10.1186/s40643-025-00968-4 (PMC12589731; doi:10.1186/s40643-025-00968-4)
Supplement: Supplementary file 2 — Supplementary Material 2 [file 40643_2025_968_MOESM2_ESM.docx]

Valorization of *Caragana korshinskii* Kom. using synthetic *Aspergillus oryzae* and *Saccharomyces cerevisiae* to produce fermented feed protein

Sasa Zuo^a^, Jing Su^a^, Fuqiang Zhang^a^, Shuying Yu^a,^ Xiaohui Cao^a^, and Chuncheng Xu*

^a^Department of Agricultural Engineering, College of Engineering, China Agricultural University, Beijing 100083, China

*Corresponding Author: Chuncheng Xu. Tel: +86-10-6273-6480, Fax: +86-10-6273-6480. E-mail address: [xucc@cau.edu.cn](mailto:xucc@cau.edu.cn).

**SUPPORTING INFORMATION**

Number of figures: 1

Number of tables: 5

Number of pages: 7

Table S1 The ratio of the diameter of transparent circle to the colony diameter and enzyme activity analysis

| Stains | D (mm) | d (mm) | D/d | Cellulase activity（U/mmol） |
| --- | --- | --- | --- | --- |
| *A.oryzae* | 50 | 25 | 2 | 35.07 |
| *E.cristatum* | 14 | 9 | 1.5 | 5.94 |
| *T. reesei* | - | - | - | 0.06 |
| *B. subtilis* | 25 | 6 | 4.2 | 10.95 |

D, diameter of strain transparent circles; d, colony diameter; D/d, ratio of the diameter of strain transparent circle to the colony diameter; -, Not tested

Table S2 Plackett-Burman (PB) experimental design and results

| Run | X_1_ (g) | X_2_ (g) | X_3_ (g) | X_4_ (g) | X_5_ (mL) | *C. korshinskii* (g) | TP (%) |
| --- | --- | --- | --- | --- | --- | --- | --- |
| 1 | -1 (1) | 1 (5) | 1 (1) | -1 (0) | 1 (2) | 4 | 7.89 |
| 2 | -1 (1) | 1 (5) | -1 (0) | 1 (2.2) | 1 (2) | 4 | 8.80 |
| 3 | -1 (1) | 1 (5) | 1 (1) | 1 (2.2) | -1 (0) | 4 | 7.58 |
| 4 | -1 (1) | -1 (1) | -1 (0) | -1 (0) | -1 (0) | 8 | 6.14 |
| 5 | 1 (5) | -1 (1) | -1 (0) | -1 (0) | 1 (2) | 4 | 7.97 |
| 6 | -1 (1) | -1 (1) | 1 (1) | -1 (0) | 1 (2) | 8 | 6.97 |
| 7 | 1 (5) | -1 (1) | 1 (1) | 1 (2.2) | 1 (2) | 4 | 7.92 |
| 8 | 1 (5) | 1 (5) | 1 (1) | -1 (0) | -1 (0) | 0 | 10.00 |
| 9 | -1 (1) | -1 (1) | -1 (0) | 1 (2.2) | -1 (0) | 8 | 6.84 |
| 10 | 1 (5) | 1 (5) | -1 (0) | 1 (2.2) | 1 (2) | 0 | 9.98 |
| 11 | 1 (5) | 1 (5) | -1 (0) | -1 (0) | -1 (0) | 0 | 10.50 |
| 12 | 1 (5) | -1 (1) | 1 (1) | 1 (2.2) | -1 (0) | 4 | 8.32 |

X_1_, bran; X_2_, cornmeal; X_3_, urea; X_4_, (NH_4_)_2_SO_4_; X_5_, nutrient salts

Table S3 Central composite design and the response for the TP content of the fermented products

| Run | Level of coding (true level) | | | TP（%） |
| --- | --- | --- | --- | --- |
|  | X_1_ Bran (g) | X_2_ Cornmeal (g) | X_3_ Ammonium sulfate (g） |  |
| 1 | 0 (2) | 0 (3) | -1.68 (0.03) | 7.05 |
| 2 | 0 (2) | 0 (3) | 0 (0.45) | 10.06 |
| 3 | -1 (1) | -1 (2) | 1 (0.7) | 8.64 |
| 4 | 0 (2) | 0 (3) | 0 (0.45) | 10.43 |
| 5 | 0 (2) | -1.68 (1.32) | 0 (0.45) | 8.28 |
| 6 | 0 (2) | 0 (3) | 0 (0.45) | 10.36 |
| 7 | 0 (2) | 1.68 (4.68) | 0 (0.45) | 10.91 |
| 8 | 0 (2) | 0 (3) | 1.68 (0.87) | 10.07 |
| 9 | 0 (3) | 0 (2) | 1 (0.7) | 9.19 |
| 10 | -1 (1) | -1 (2) | -1 (0.2) | 8.51 |
| 11 | 0 (2) | 0 (3) | 0 (0.45) | 10.88 |
| 12 | 1 (3) | 1 (4) | 1 (0.7) | 11.55 |
| 13 | -1.68 (0.32) | 0 (3) | 0 (0.45) | 8.00 |
| 14 | -1 (1) | 1 (4) | 1 (0.7) | 9.40 |
| 15 | 1.68 (3.68) | 0 (3) | 0 (0.45) | 9.04 |
| 16 | 1 (3) | -1 (2) | -1 (0.2) | 7.89 |
| 17 | 1 (3) | 1 (4) | -1 (0.2) | 8.35 |
| 18 | 0 (2) | 0 (3) | 0 (0.45) | 10.37 |
| 19 | 0 (2) | 0 (3) | 0 (0.45) | 10.96 |
| 20 | -1 (1) | 1 (4) | -1 (0.2) | 8.54 |

X_1_, Bran; X_2_, Cornmeal; X_3_, Ammonium sulfate

Table S4 ANOVA for response surface quadratic model by CCD

| Source of error | Sum of squares | Degree of freedom | Mean square | F-value | *P*-value |
| --- | --- | --- | --- | --- | --- |
| Model | 28.29 | 9 | 3.14 | 21.86 | ~~<0.0001~~<0.01 |
| Bran, X_1_ | 0.97 | 1 | 0.97 | 6.74 | 0.027~~66~~ |
| Cornmeal, X_2_ | 4.73 | 1 | 4.73 | 32.86 | ~~0.0002~~<0.01 |
| (NH_4_)_2_SO_4_, X_3_ | 8.18 | 1 | 8.18 | 56.89 | ~~<0.0001~~<0.01 |
| X_1_X_2_ | 0.51 | 1 | 0.51 | 3.58 | 0.088~~77~~ |
| X_1_X_3_ | 1.54 | 1 | 1.54 | 10.71 | ~~0.0084~~<0.01 |
| X_2_X_3_ | 0.86 | 1 | 0.86 | 6.01 | 0.034~~1~~ |
| X_1_^2^ | 6.24 | 1 | 6.24 | 43.42 | ~~<0.0001~~<0.01 |
| X_2_^2^ | 1.11 | 1 | 1.11 | 7.75 | 0.019~~3~~ |
| X_3_^2^ | 5.98 | 1 | 5.98 | 41.57 | ~~<0.0001~~<0.01 |
| Residual | 1.44 | 10 | 0.14~~38~~ |  |  |
| Lack of fit | 0.85 | 5 | 0.17~~695~~ | 1.44 | 0.35~~07~~ |
| Std. Dev. | 0.38 |  |  | *R^2^* | 0.952~~16~~ |
| Mean | 9.42 |  |  | Adjusted *R^2^* | 0.908~~1~~ |
| C.V.% | 4.02 |  |  | Predicted *R^2^* | 0.746~~1~~ |
|  |  |  |  | Adeq Precision | 15.64~~371~~ |

~~Table S5 Overview of DIA protein identification~~

| **~~Name~~** | **~~Peptide~~** | **~~Identified protein~~** |
| --- | --- | --- |
| ~~ALL~~ | ~~69892~~ | ~~8011~~ |

Table S5 Number of differentially expressed proteins across treatment groups

| Comparison | Total | Up-regulated | Down-regulated | No-significant | Unique Proteins in Aor | Unique Proteins in Sce | Unique Proteins in MixF |
| --- | --- | --- | --- | --- | --- | --- | --- |
| Aor. vs. Sce | 6395 | 5004 | 1137 | 254 | 4875 | 845 | -- |
| MixF. vs. Sce | 6024 | 4649 | 1109 | 266 | -- | 309 | 4543 |
| MixF. vs. Aor | 6252 | 212 | 79 | 5961 | 12 | -- | 127 |

Aor, the substrate treated with *Aspergillus oryzae*; Sce, the substrate treated with *Saccharomyces cerevisiae*; MixF, the substrate treated with mixed *A. oryzae* and *S. cerevisiae*

**Figure S1.** Proteomics analysis. Total sample principal component analysis (a), Venn diagram of functional annotation results (b), differential protein volcano plot and KEGG enriched bubble chart between MixF and Sce (c and d), and chord diagrams of Go-enriched and KEGG-enriched differentially expressed proteins in the MixF versus Aor group. GO, KOG, KEGG, IPR, and Subcellular, functional annotation database. CK, only sterilized substrates; Sce, *Saccharomyces cerevisiae*, Aor, *Aspergillus oryzae*, and MixF, the mixed *S. cerevisiae* and *A. oryzae* fermented substrates


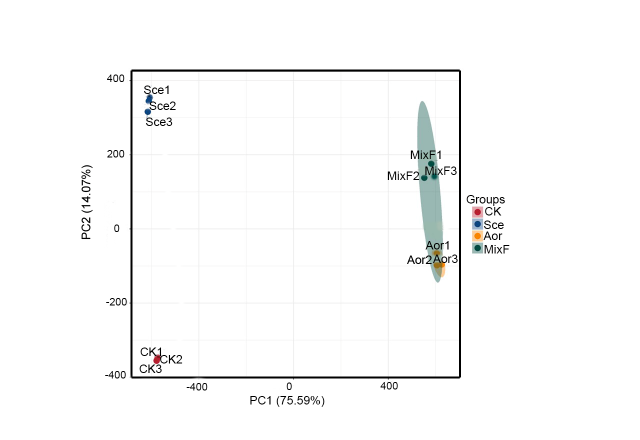


a


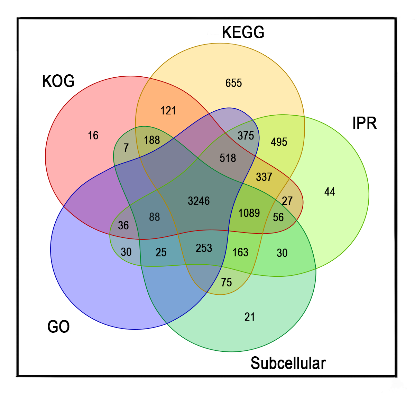


b


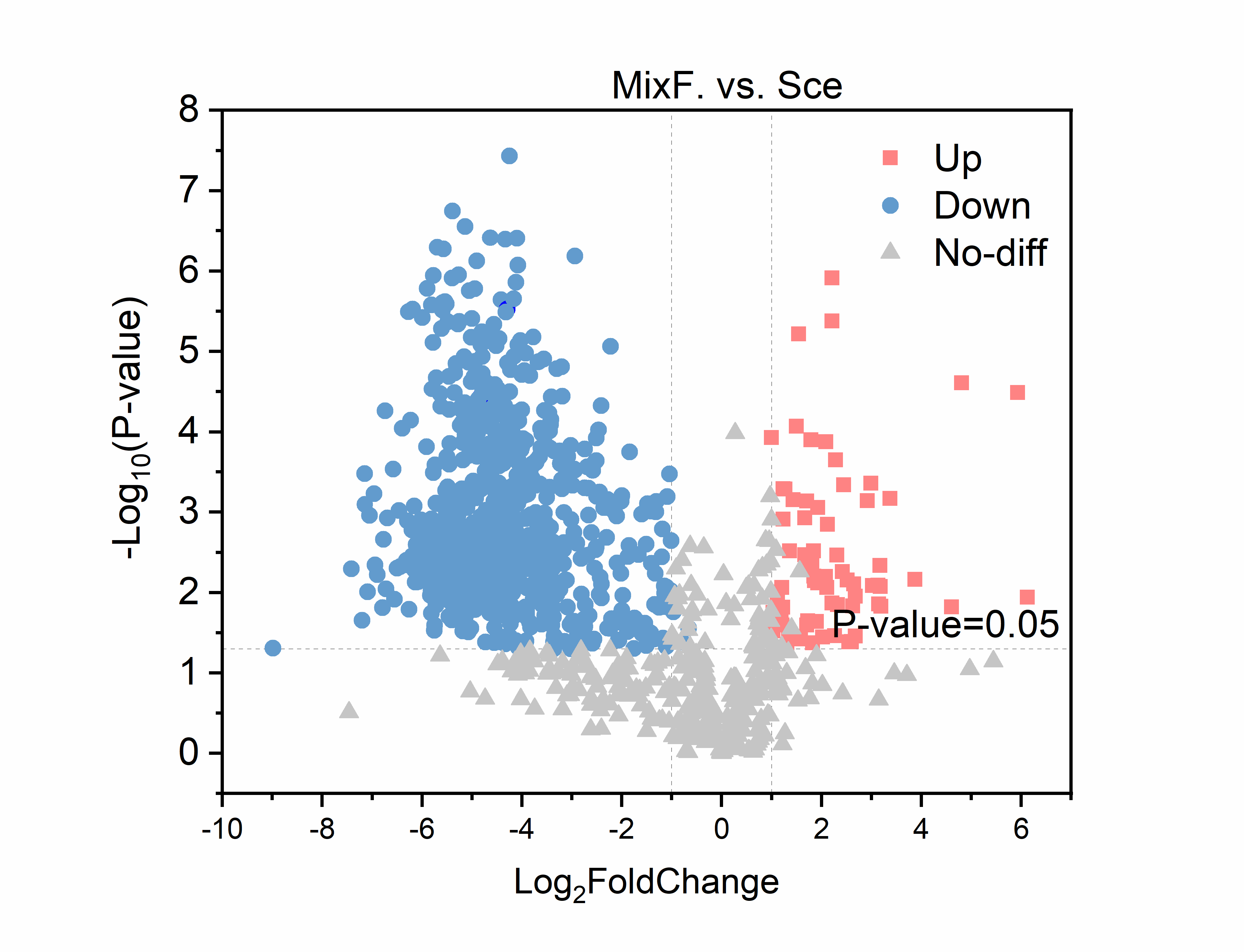


c


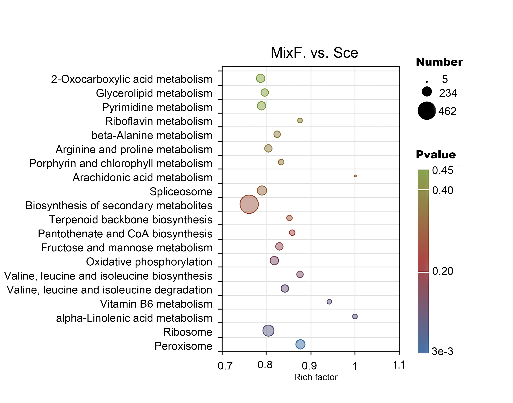


d


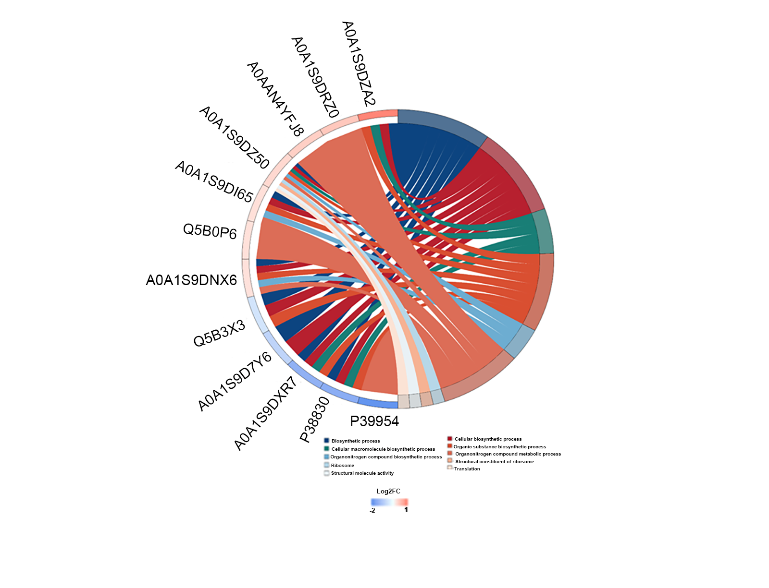


e


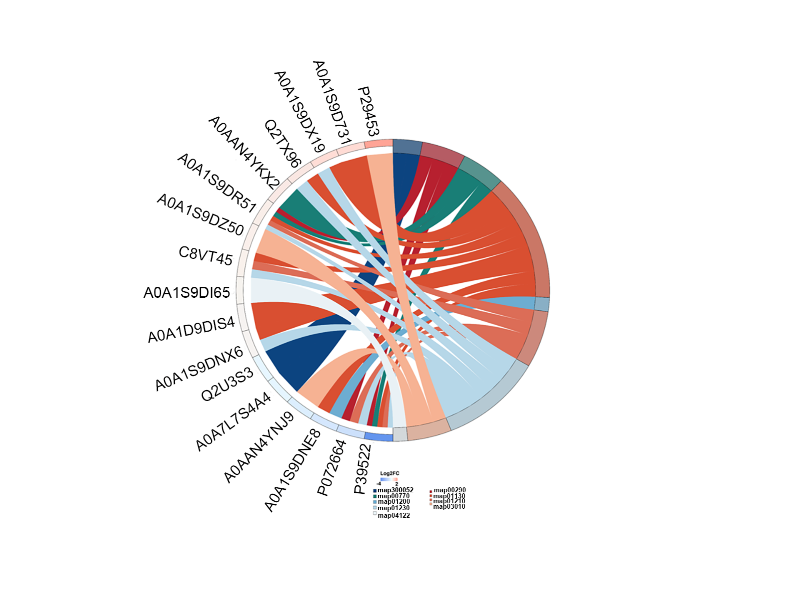


f
